# The Lower Concentration of Plasma Acetyl-Carnitine in Epicardial Artery Disease—A Preliminary Report

**DOI:** 10.3390/ijms26031318

**Published:** 2025-02-04

**Authors:** Tomasz Urbanowicz, Paweł Gutaj, Szymon Plewa, Anna Olasińska-Wiśniewska, Ievgen Spasenenko, Beata Krasińska, Andrzej Tykarski, Krzysztof J. Filipiak, Martyna Pakuła-Iwańska, Zbigniew Krasiński, Ewelina Grywalska, Ewa Wender-Ożegowska, Marek Jemielity, Jan Matysiak

**Affiliations:** 1 Cardiac Surgery and Transplantology Department, Poznan University of Medical Sciences, 61-701 Poznan, Poland; 2 Department of Reproduction, Poznan University of Medical Sciences, 61-701 Poznan, Poland; 3 Department of Inorganic and Analytical Chemistry, Faculty of Pharmacy, Poznan University of Medical Sciences, 61-701 Poznan, Poland; 4 Department of Hypertensiology, Angiology and Internal Medicine, University of Medical Sciences, 61-701 Poznan, Poland; 5 Institute of Clinical Science, Maria Sklodowska-Curie Medical Academy, 00-136 Warsaw, Poland; 6 Department of Vascular, Endovascular Surgery, Angiology and Phlebology Medical University, Poznan University of Medical Science, 61-701 Poznan, Poland; 7 Department of Experimental Immunology, Medical University of Lublin, Chodzki Street, 20-059 Lublin, Poland

**Keywords:** acetyl-carnitine, coronary artery disease, metabolomic, coronary atherosclerosis

## Abstract

Coronary artery disease remains an epidemiological challenge as global morbidity is not declining despite the fact that the risk factors are well known. Metabolomic derivatives of atherosclerosis formation have recently gained attention as a possible non-traditional risk factor. The aim of this study was to find potential differences in acetyl-carnitine chain serum concentrations between epicardial artery disease patients and a control group. There were 41 patients (25 men and 16 women), with a median (Q1–Q3) age of 69 (63–73) years, enrolled in the prospective metabolomic analysis. They were divided into two groups based on cine angiography results confirming epicardial artery disease (group 1, *n* = 25 (61%)) or showing characteristics corresponding to normal angiograms (group 2, *n* = 16 (39%)). The quantitation of metabolites was performed based on the coronary angiograms. Significant differences related to the plasma concentration of L-Acetyl-carnitine (7.49 (4.79–9.23) µM vs. 9.36 (8.57–10.23) µM (*p* = 0.009)), Decanoyl-carnitine (0.00 (0.00–0.37) µM vs. 0.36 (0.19–0.44) µM (*p* = 0.040)), C12:1-carnitine (0.17 (0.14–0.20) µM vs. 0.22 (0.18–0.24) µM (*p* = 0.008)), trans-2-Dodecenoyl-carnitine (0.10 (0.07–0.13) µM vs. 0.13 (0.10–0.15) µM (*p* = 0.002)), cis-5-Tetradecenoyl-carnitine (0.03 (0.02–0.04) µM vs. 0.04 (0.03–0.05) µM (*p* = 0.043)), and 3,5-Tetradecadien-carnitine (0.16 (0.14–0.18) µM vs. 0.18 (0.17–0.27) µM (*p* = 0.007)) in group 1 vs. group 2 were noted. Increased plasma levels of acetyl-carnitine may be characteristic of patients with normal coronary angiograms.

## 1. Introduction

Coronary artery disease (CAD) remains an epidemiological challenge, as global morbidity is not declining even though the risk factors are well-known [1]. The genetic factors, along with environment and lifestyle, are linked with an increased risk of cardiovascular disease development [2]. There is a high prevalence of subclinical epicardial disease in the general population despite proposed screening tests and improved risk stratification [3]. The cornerstone for effective prevention is properly assessing atherosclerotic cardiovascular disease threats [4]. Non-traditional risk factors are presently being thoroughly investigated to improve long-term outcomes.

As cardiovascular disease jeopardy prediction is an under-addressed clinical subject, exploring novel prognostic factors, including metabolic biomarkers, is raising interest. Atherosclerotic disease is considered a chronic inflammatory condition [5,6,7], for which anti-inflammatory therapeutic approaches have recently gained attention [8,9].

A marked heterogeneous presentation, including chronic and acute stages and various forms between them, characterizes CAD. While classical markers, such as troponin or brain natriuretic peptide (BNP), are useful for patients with heart dysfunction, metabolomic molecules have recently gathered even more interest as experimental features of impaired metabolic flexibility. The early detection of markers associated with an increased risk of cardiovascular events would be crucial in clinical practice. Lv et al. [10] suggested adding metabolomic parameters to the tailed risk assessment of acute cardiovascular events. Middle- and long-chain acetyl-carnitines have been identified as being associated with cardiovascular death, myocardial infarction, and stroke. Among the naturally occurring amino acids characterized by anti-inflammatory effects is L-carnitine, which is believed to influence the cardiovascular system considerably [11]. Previous studies [11] suggested l-carnitine (LC) supplementation had a beneficial effect on oxidative stress and inflammatory biomarkers in patients with CAD. Gander et al. [12] indicated a mitochondrial imbalance exists in patients with coronary artery disease based on altered acetyl-carnitine levels. A relationship between short-chain acetyl-carnitine serum concentration and heart failure was suggested in the DEFINE-HF (Dapagliflozin Effects on Biomarkers, Symptoms and Functional Status in Patients with HF with Reduced Ejection Fraction) trial [13]. The influence of obesity and dietary habits on plasma acetyl-carnitine and heart failure risk was reported by France et al. [14]. The role of specific metabolites in the metabolic pathways and their differences in various disease presentations is still unclear.

The aim of the study was to find potential differences in acetyl-carnitine chain serum concentrations between epicardial artery disease patients and a control group.

## 2. Results

Patients were divided into two groups based on cine angiography results confirming epicardial artery disease (group 1, *n* = 25 (61%)) or resulting exhibiting characteristics corresponding to normal angiograms (group 2, *n* = 16 (39%)). The demographic and clinical characteristics are presented in Table 1.

The cine-angiography, echocardiographic, and laboratory test results were compared between both groups, as presented in Table 2.

A metabolomic evaluation of carnitine in the analyzed population was performed, and the results were compared between groups. The 20 types of acetyl-carnitine plasma concentrations were investigated.

The following explored parameters were not incorporated into the analysis as they did not reach the LOD value: Hydroxypropionyl-carnitine, Propenoyl-carnitine, 3-Methylglutaryl-carnitine, Tiglyl-carnitine, Glutaconyl-carnitine, 2-Hexenoyl-carnitine, 2,6 Dimethylheptanoyl-carnitine/Nonanoyl-carnitine, Dodecanoyl-carnitine, Dodecanedioylcarnitine, Tetradecanoylcarnitine, 3-Hydroxy-cis-5-tetradecenoylcarnitine, Palmitoyl-carnitine, 3-Hydroxyhexadecanoyl-carnitine, trans-Hexadec-2-enoyl-carnitine/9-Hexadecenoyl-carnitine, 3-Hydroxy-9-hexadecenoy-lcarnitine, 9,12-Hexadecadienoyl-carnitine, 3-Hydroxyhexadecadienoyl-carnitine, Stearoyl-carnitine, and 3-Hydroxy-11Z-octadecenoyl-carnitine. As only one positive sample of 9-Decenoyl-carnitine and two positive samples of Hexanoylcarnitine were obtained, these parameters were not considered. The plasma acetyl-carnitine chains that were incorporated into the analysis are presented in Table 3.

**Figure 1:**
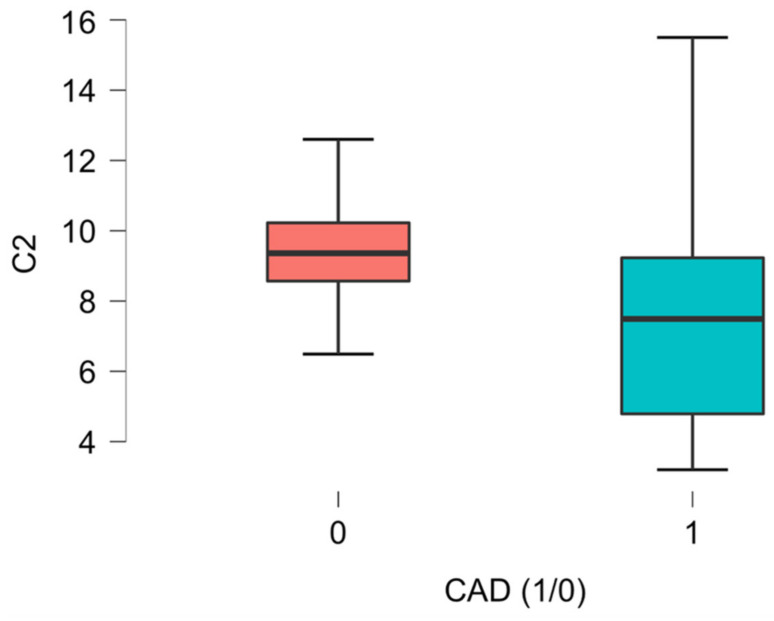
C2 acetyl-carnitine (L-Acetyl-carnitine) differences in plasma concentration [uM] between CAD group (1) vs non-CAD group (0) (*p* = 0.009).

**Figure 2:**
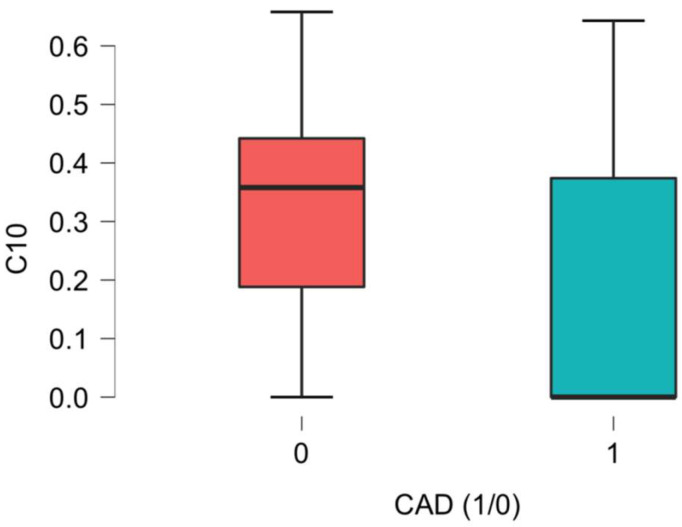
C10 acetyl-carnitine (Decanoyl-carnitine) differences in plasma concentration [uM] between CAD group (1) vs non-CAD group (0) (*p* = 0.040).

**Figure 3:**
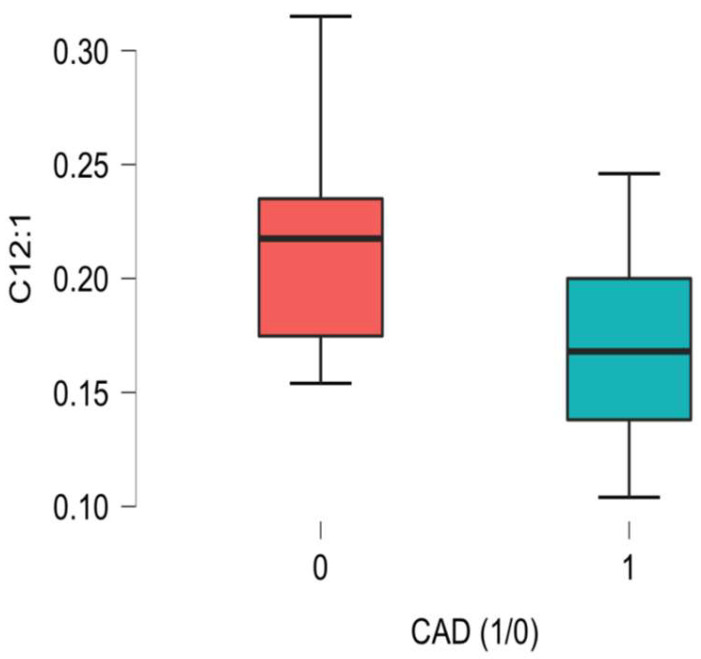
C12:1 acetyl-carnitine (trans-2-Dodecenoyl-carnitine) differences in plasma concentration [uM] between CAD group (1) vs non-CAD group (0) (*p* = 0.008).

**Figure 4:**
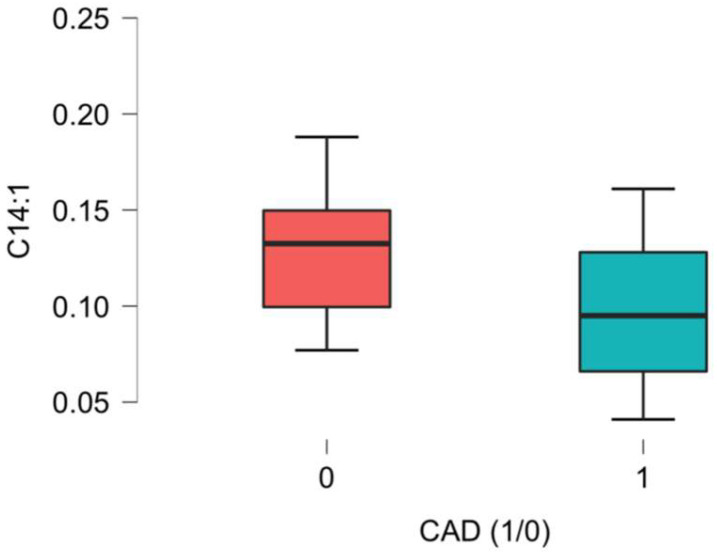
C14:1 acetyl-carnitine (cis-5-Tetradecenoyl-carnitine) differences in plasma concentration [uM] between CAD group (1) vs non-CAD group (0) (*p* = 0.002).

**Figure 5:**
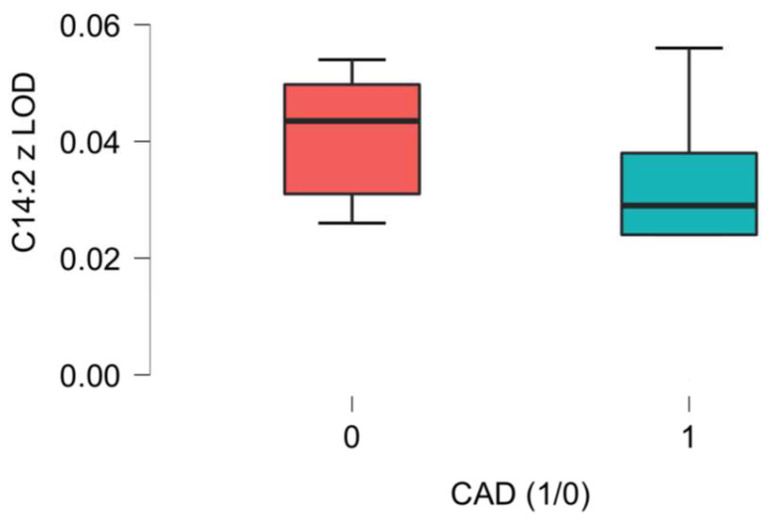
C14:2 acetylcarnitine (3, 5-Tetradecadien-carnitine) differences in plasma concentration [uM] between CAD group (1) vs non-CAD group (0) (*p* = 0.043).

**Figure 6:**
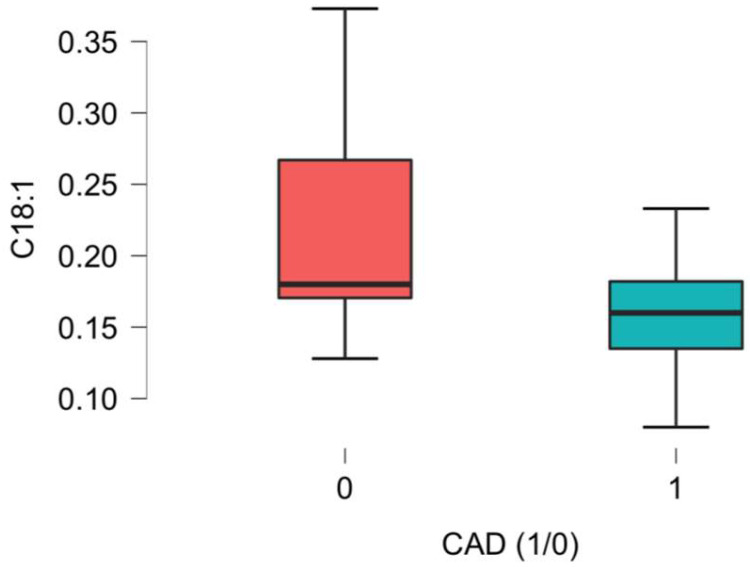
C18:1 acetyl-carnitine (Oleoyl-carnitine/Vaccenyl-carnitine/Elaidic-carnitine) differences in plasma concentration [uM] between CAD group (1) vs non-CAD group (0) (*p* = 0.007).

Significant differences related to the plasma concentrations of L-Acetylcarnitine between the CAD group and the non-CAD group were noted, as presented in Figure 1, Figure 2, Figure 3, Figure 4, Figure 5 and Figure 6.

In the analyzed groups, we found significant differences regarding high-density lipoprotein (*p* = 0.010) but not total cholesterol (*p* = 0.466), low-density lipoprotein (*p* = 0.762), or triglycerides (*p* = 0.803).

**Figure 7:**
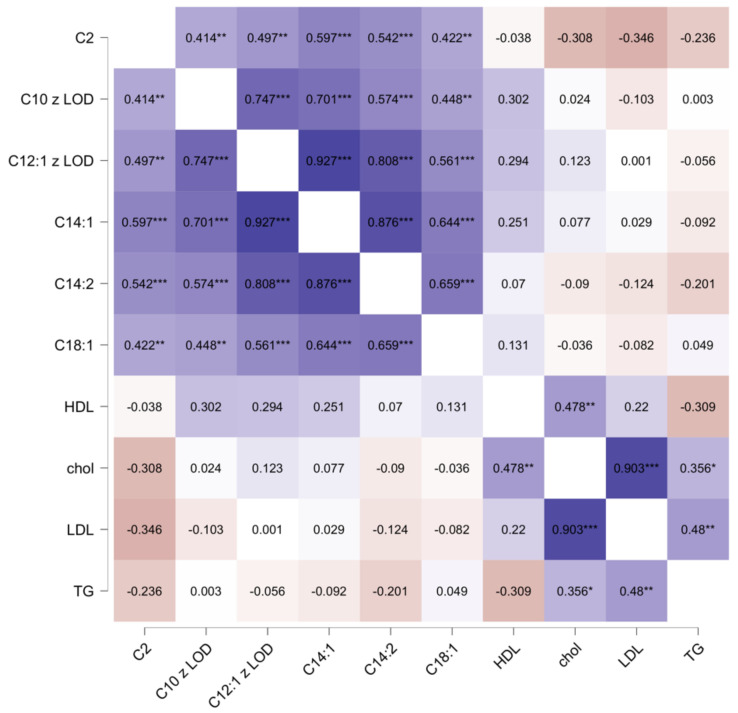
Possible correlations between numerous acetyl-carnitines in the CAD group. Velvet and peach colors correspond to positive and negative correlation coefficients, respectively. The saturation of each color reflects the absolute value of the correlation coefficient. *—*p* < 0.050, **—*p* < 0.010, and ***—*p* < 0.001. Abbreviations: C2—L-Acetylcarnitine; C10—Decanoylcarnitine; C12:1—trans-2-Dodecenoylcarnitine; C14:1—cis-5-Tetradecenoylcarnitine; C14:2—3,5-Tetradecadiencarnitine; C18:1—Oleoylcarnitine/Vaccenyl carnitine/Elaidic carnitine; chol—total cholesterol; HDL—high-density lipoprotein; LDL—low-density lipoprotein; TG—triglycerides.

We noticed a positive correlation between various acetyl-carnitine types but not lipidogram components, including HDL, as presented in Figure 7.

As males are predisposed to developing CAD disease, we compared the acetyl-carnitine results related to the male sex via rank-biserial correlation, as presented in Table 4. The results revealed significant sex-related differences for CAD predisposition related to L-Acetyl-carnitine (*p* = 0.036), Decanoyl-carnitine (*p* = 0.003), trans-2-Dodecenoyl-carnitine (*p* = 0.10), cis-5-Tetradecenoyl-carnitine (*p* = 0.011), and Oleoyl-carnitine/Vaccenyl-carnitine/Elaidic-carnitine (*p* = 0.035), as presented in Table 4.

## 3. Discussion

The results of our analysis point out the significantly lower plasma concentrations of low-chain acetyl-carnitine in coronary artery patients. The presented decreases were found to be independent of the lipogram results. The suggested scenario of atherosclerosis development may be regarded as unrelated to lipid-dependent pathways.

Our study enables an understanding of the molecular aspects involved in different clinical presentations of cardiovascular diseases. Our study population included patients with stable coronary artery disease and controls; both groups were burdened with the classical cardiovascular risk. However, the metabolomic profiles differed between them.

Carnitine is a naturally occurring amino acid required to transport long-chain fatty acids into mitochondria, indicating its significant function in fat metabolism [15]. In animal studies [16], it has been shown that metabolic reprogramming via abolishing the fatty acid oxidation of cardiomyocytes may improve their resistance to hypoxia and stimulate cardiomyocyte proliferation, allowing heart regeneration. The anti-inflammatory properties of carnitine, combined with its antioxidant function as a scavenger of free radicals, were suggested in previous studies [17]. In randomized studies, carnitine was shown to be beneficial by increasing antioxidant enzyme activities and reducing oxidative stress [18].

Our analysis revealed significantly lower plasma concentrations of C2, an acetylated derivative of the amino acid L-carnitine whose function is related to energy metabolism regulation within mammalian mitochondria [19]. This acetic acid ester of carnitine plays a role in fatty acid metabolism by accelerating acetyl-CoA into the matrices of mitochondria during the oxidation of fatty acids [20]. The role of acid L-carnitine in neuropathophysiology has been widely reported [21,22], but its relation to coronary artery disease is inconclusive [23,24].

Significant differences between the CAD and non-CAD groups regarding sC10, which stands for decanoyl-carnitine, were noted in our report. It is a decanoic acid ester of carnitine. It is a medium-chain acylcarnitine [19]. It is formed either through esterification with L-carnitine or through the peroxisomal metabolism of longer-chain acylcarnitines [25]. It has been proposed to be a useful marker for inherited fatty acid metabolism disorders, especially for hypertensive patients [26].

A higher concentration of the trans-2-dodecenoic acid ester of carnitine, an acetyl-carnitine denoted as C12:1, in the control group was reported in our analysis. This medium-chain acylcarnitine trans-2-dodecenoyl-carnitine’s role in pathophysiology is not very well known, though previous reports have indicated its possible role in oncology and inflammatory processes [27,28].

The other acetyl-carnitine that differentiated our groups was cis-5-Tetradecenoyl-carnitine, listed as C14:1. It is classified as a long-chain AC and forms through esterification with fatty acids obtained from one’s diet. Increased plasma levels have also been observed in patients who have lost weight [29]. A higher plasma content was characteristic of non-coronary-disease patients in our study.

A higher plasma concentration of 3,5-Tetradecadien-carnitine, denoted as C14:2, was noted in our analysis. This is another long-chain AC who’s main function is related to the transport of fatty acids into the mitochondria [30]. Our analysis brings a new perspective on the possible role of this long-chain AC in cardiovascular pathology.

Oleoyl-carnitine, Vaccenyl-carnitine, and Elaidic-carnitine are long-chain carnitines whose concentrations in plasma are diet-dependent. Fatty acid derivative accumulation was postulated to play a role in Vaccenyl-carnitine deficiency, representing one of the most common inherited lipid metabolism disorders [31]. Elastic carnitine deficiency was reported to cause fatal effects related to profound disturbances in fatty acid oxygenation [32].

Our metabolomics analysis suggests a new role of acetyl-carnitine short-, medium-, and long-chain esters arising from the conjugation of acyl chains to the hydroxyl group of L-carnitine in the possible pathophysiology of coronary artery disease (besides lipid disturbances). The sex-related differences in CAD prevalence were also confirmed via acyl-carnitine concentrations, which may suggest the fairness of our hypothesis.

Previous studies have reported altered levels of acetyl-carnitines in patients with myocardial injury, reflected in heart failure with a reduced ejection fraction [33], and a decrease in patients treated with mechanical support [34]. We examined a study population burdened with a significant cardiovascular risk. Patients treated pharmacologically who presented with a stable form of coronary artery disease had lower levels of acetyl-carnitines, which may shed light on the impact of the disease stage on metabolomic profiles. Further, larger-scale studies comparing metabolomic markers in patients with different stages of coronary artery disease with and without heart failure are necessary.

## 4. Study Limitations

This study was performed on a limited number of patients who corresponded to the typical clinical scenario for coronary artery disease, with a prevalence of the male sex. The results were compared with chronic coronary syndrome patients and normal cine angiograms. Due to the limited number of participants, the preliminary report indicated the differences between the coronary and non-coronary disease populations were related to acetyl-carnitine serum concentrations but not other potential factors. The insignificant differences regarding the clinical data suggest a possible novel epicardial artery atherosclerotic plaque formation pathway.

## 5. Methods

### 5.1. Patients

**Figure 8:**
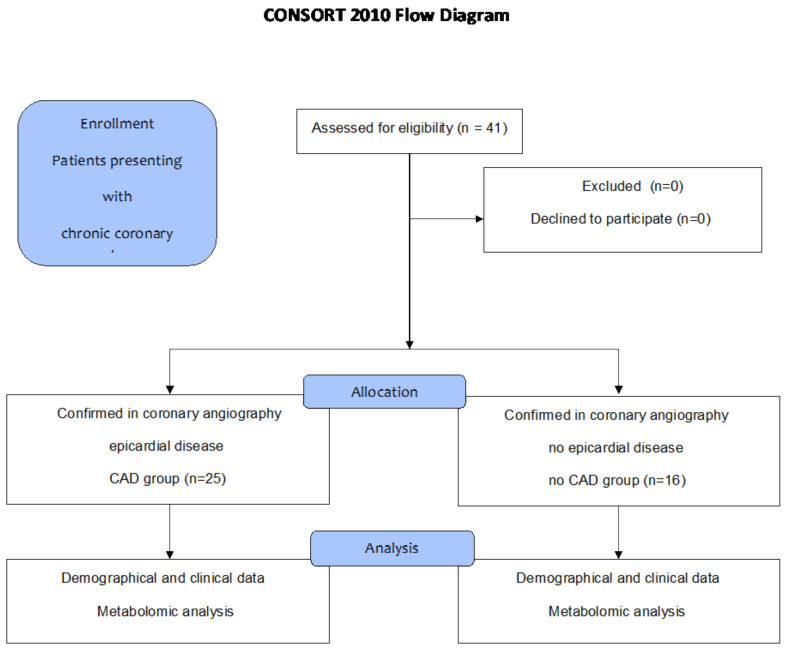
Study flowchart. Abbreviations: CAD—coronary artery disease; *n*—number.

A total of 41 consecutive patients (25 men and 16 women), with a median (Q1–Q3) age of 69 (63–73) years, were enrolled in the prospective metabolomic analysis. They presented with chronic coronary syndrome (CCS) class (2 ± 0.3) and were hospitalized in the internal medicine and hypertension department from 2022 to 2024. To present the real-life results for a limited number of participants, consecutive patients admitted for coronary angiograms were incorporated into the analysis. Patients with inflammatory and neoplasm diseases were excluded. Clinical and demographic data were recorded. The co-morbidities in the presented group included arterial hypertension (33 (81%)) and dyslipidemia (33 (81%)), followed by diabetes mellitus (11 (27%)), chronic obstructive pulmonary disease (6 (15%)), kidney dysfunction (4 (10%)), and peripheral artery disease (3(7%)). Patients were divided into two groups based on cine angiography results confirming epicardial artery disease (CAD group 1, *n* = 25 (61%)) or showing characteristics corresponding to normal angiograms (non-CAD group 2, *n* = 16 (39%)) (Figure 8).

A clinical evaluation was performed during hospitalization, followed by transthoracic echocardiography, cine-angiography, and laboratory tests. Additionally, peripheral blood samples were taken for metabolomics analyses.

### 5.2. Methods

The methods employed included laboratory data analysis comprising whole blood count parameters using a routine hematology analyzer (Sysmex Europe GmbH, Norderstedt, Germany).

Anthropometric measurements were obtained using a Marsden M-100 Column Scale with Height Measure (Charder Electronics, Co. Ltd., London, UK).

The quantitation of metabolites was performed using the AbsoluteIDQ p180 kit (Biocrates LifeSciences AG, Innsbruck, Austria). All compounds were analyzed using a single sample preparation procedure. For single sample determination, the 10 µL of serum was used. The samples were prepared according to the manufacturer’s instructions. The samples were analyzed in a blind manner in a random order. The filter layer, pre-pipetted by the manufacturer, contained stable isotope labeled internal standards (ISs). The remaining ISs were prepared as per the manufacturer’s guidelines and pipetted onto a kit plate. Then, calibrators and quality control (QC) samples were prepared and put onto a plate. Then, 10 μL aliquots of thawed serum samples were pipetted onto the filter layer of a 96-deep well plate, and the plate was dried under nitrogen flow for 30 min. After that, the freshly prepared derivatization solution was pipetted onto the plate and incubated under a cover for 25 min. Then, the plate was dried under nitrogen flow (60 min). The extraction solvent was added to each well in the next step, and the plate was shaken for 30 min (450 rpm). The extracts were further pushed through the filter layer using nitrogen flow. The obtained extracts were divided into two separate 96-well plates and diluted according to the manufacturer’s specifications. After being sealed with silicone naps, the plates were put into a thermostatic autosampler.

The metabolites were analyzed using a liquid chromatography–tandem mass spectrometry system (LC-MS/MS) that combines a 1260 Infinity HPLC (Agilent Technologies, Santa Clara, CA, USA) liquid chromatograph with a 4000QTRAP mass spectrometer (SCIEX, Framingham, MA, USA). The compounds were analyzed using flow injection analysis (FIA-MS/MS method). Two groups of chemical compounds, which have already been covered in the Methods Section (amino acids and biogenic amines), were separated prior to detection by the use of a ZORBAX Eclipse XDB-C18 (3.0 × 100 mm, 3.5 μm) column (Agilent Technologies, Santa Clara, CA, USA), with a pre-column (C18, 4.0 × 3.0 mm) SecurityGuard (Phenomenex, Torrance, CA, USA) and mass spectrometry detection and quantitation.

The data acquisition process was performed using Analyst 1.6.3 software (Sciex, Framingham, MA, USA). Data processing, integration, calibration, and evaluation were performed using WebIDQ software (Biocrates LifeSciences AG, Innsbruck, Austria).

### 5.3. Statistical Analysis

The Shapiro–Wilk test was used to assess the normality of data distribution. According to the test result, clinical and biochemical continuous variables were presented as medians and interquartile ranges [Q1–Q3] and compared using the non-parametric Mann–Whitney test. Where applicable, categorical data were expressed as numbers and percentages and compared using Fisher’s exact test or Chi-square test. Spearman correlation analysis was used to describe the correlation between the variables. GPower (version 3.1.9.2; Institute for Experimental Psychology in Dusseldorf, Germany) testing was applied to calculate the sample size. Based on the assumption of an undetermined target population size, with α = 0.05 and β = 0.2, the minimum sample size was estimated to be 24 participants. Hence, we decided to collect complete data from at least 25 participants and a roughly equivalent number of controls.

To compare the results obtained for the CAD male population with the rest of the analyzed groups, a rank-share real correlation with 95% confidence intervals was applied. Statistical analysis was performed using JASP software [JASP Team; 2023. Version 0.18.1]. *p* < 0.05 was considered statistically significant (https://jasp-stats.org, accessed on 15 December 2024).

### 5.4. Bioethics Committee

The Institutional Ethics Committee of Poznan University of Medical Sciences, Poznan, Poland (protocol code 694/20 on 4 November 2020), approved this study, which was conducted in accordance with the principles outlined in the Declaration of Helsinki.

## 6. Conclusions

Increased plasma levels of acetyl-carnitine may characterize patients with normal coronary angiograms. In our preliminary report, we noticed significantly lower plasma levels of L-Acetyl-carnitine, Decanoyl-carnitine C12:1, trans-2-Dodecenoyl-carnitine, cis-5-Tetradecenoyl-carnitine, and 3,5-Tetradecadien-carnitine in patients presenting with angiography-proven coronary disease. The metabolomic results could indicate a novel, non-lipid-dependent pathway of coronary atherosclerosis development. Further studies are required to confirm the suggested hypothesis.

## Figures and Tables

**Table 1:** Demographic and clinical characteristics.

Parameters	Whole Group *n* = 41	Group 1 CAD Group *n* = 25	Group 2 Non-CAD Group *n* = 16	*p* Group 1 vs. 2
Demographics				
Age (years) (median (Q1–Q3))	69 (63–73)	69 (63–73)	70 (64–74)	0.556
Height (cm) (median (Q1–Q3))	165 (163–176)	167 (164–176)	164 (162–176)	0.628
Weight (kg) (median (Q1–Q3))	80 (70–91)	80 (71–92)	80 (67–88)	0.645
BMI (median (Q1–Q3))	28.6 (25.4–31.8)	29.1 (25.4–30.9)	28.2 (25.5–32.3)	0.822
BMI > 30	17 (41)	10 (40)	7 (44)	1.000
Clinical status:				
CCS class				
class I (*n* (%))	1 (2)	0 (0)	1 (6)	
class II (*n* (%))	34 (83)	19 (76)	15 (94)	0.056
class III (*n* (%))	6 15	6 (24)	0 (0)	
NYHA class				
class I (*n* (%))	23 (56)	13 (52)	10 (63)	
class II (*n* (%))	17 (42)	11 (44)	6 (37)	0.628
class III (*n* (%))	1 (2)	1 (4)	0 (0)	
Co-morbidities:				
Arterial hypertension (*n* (%))	35 (85)	22 (88)	13 (81)	0.662
Diabetes mellitus (*n* (%))	12 (29)	7 (28)	5 (31)	1.000
Dyslipidemia (*n* (%))	33 (83)	21 (84)	13 (81)	1.000
COPD (*n* (%))	7 (17)	5 (20)	2 (13)	0.685
Kidney dysfunction * (*n* (%))	5 (12)	3 (12)	2 (13)	1.000
PAD (*n* (%))	4 (10)	3 (12)	1 (6)	1.000
Thyroid disease (*n* (%))	6 (15)	2 (8)	4 (25)	0.187
Atrial fibrillation (*n* (%))	5 (12)	2 (8)	3 (19)	0.362

Abbreviations: BMI—body mass index, CAD—coronary artery disease, CCS—Canadian Cardiovascular Society, cm—centimeter, COPD—chronic obstructive pulmonary disease, kg—kilogram, *n*—number, PAD—peripheral arterial disease, and Q—quartiles.

**Table 2:** Cine angiography, echocardiographic, and laboratory results.

Parameters	Whole Group *n* = 41	Group 1 CAD Group *n* = 25	Group 2 Non-CAD Group *n* = 16	*p* Group 1 vs. 2
Cine-angiography results:				
Normal angiograms (*n* (%))	16 (39)	0 (0)	16 (100)	-
Epicardial disease	25 (61)	25 (100)	0 (0)	-
LMCA (*n* (%))	5 (12)	5 (20)	0 (0)	-
One-vessel (*n* (%))	2 (5)	2 (8)	0 (0)	-
Two-vessel (*n* (%))	10 (24)	10 (40)	0 (0)	-
Three-vessel disease (*n* (%))	8 (20)	8 (32)	0 (0)	-
Echocardiographic results:				
LVEDD (mm) (median (Q1–Q3))	48 (44–52)	49 (46–52)	44 (42–50)	0.117
RVEDD (mm) (median (Q1–Q3))	29 (27–31)	29 (27–31)	29 (28–31)	0.958
LAD (mm) (median (Q1–Q3))	36 (33–39)	37 (34–39)	34 (30–38)	0.537
IVS (mm) (median (Q1–Q3))	12 (10–13)	12 (11–13)	12 (10–13)	0.494
LVEF (%) (median (Q1–Q3))	55 (50–60)	50 (50–60)	60 (52–62)	0.309
LV contractility disturbances (*n* (%))	24 (59)	15 (60)	9 (56)	1.000
IM (grade) mild vs. moderate-severe (*n* (%))	21 (51)/8 (20)	10 (40)/5 (20)	11 (69)/3 (19)	0.682
IT (grade) mild vs. moderate (*n* (%))	30 (73)/5 (12)	16 (64)/3 (12)	14 (88)/2 (13)	1.000
Laboratory tests:				
Whole blood count analysis				
WBC (109/L) (median (Q1–Q3))	6.98 (6.03–8.02)	7.51 (6.47–8.86)	6.79 (5.68–7.41)	0.073
Hb (mmol/L) (median (Q1–Q3))	8.9 (8.20–9.40)	9.20 (8.40–9.70)	8.50 (8.00–9.00)	0.051
Hct (%) (median (Q1–Q3))	43 (41–45)	44 (41–46)	41 (40.5–43.3)	0.104
Plt (109/L) (median (Q1–Q3))	221 (196–264)	226 (209–264)	221 (180–253)	0.479
NLR	2.63 (2.09–3.45)	2.86 (2.46–3.90)	2.09 (1.81–2.66)	0.009
MLR	0.29 (0.24–0.37)	0.28 (0.23–0.36)	0.30 (0.26–0.35)	0.680
C-reactive protein (mg/L)	8 (7–9)	8 (7–9)	8 (7–9)	0.554
Kidney function tests				
Creatinine (mmol/L) (median (Q1–Q3))	81.0 (73.0–91.0)	83 (73–101)	77.5 (71.3–89.5)	0.454
Liver function tests				
ALT (IU/L) (median (Q1–Q3))	29 (24.5–38.5)	31 (26–42)	27.5 (22–33)	0.241
Lipidogram				
Total cholesterol (mmol/L) (median (Q1–Q3))	4.16 (3.52–4.70)	3.83 (3.18–4.69)	4.37 (3.83–4.76)	0.235
LDL (mmol/L) (median (Q1–Q3))	2.43 (1.67–3.10)	2.45 (1.76–3.17)	2.16 (1.697–2.92)	0.652
HDL (mmol/L) (median (Q1–Q3))	1.22 (1.01–1.56)	1.09 (0.91–1.28)	1.57 (1.33–1.71)	0.010
Triglycerides (mmol/L) (median (Q1–Q3))	1.30 (0.94–1.79)	1.28 (1.00–1.78)	1.39 (0.90–1.79)	0.825
TSH (mIU/L)	2.5 (1.7–3.2)	2.5 (1.6–3.2)	2.6 (1.8–3.1)	0.911

Abbreviations: ALT—alanine transaminase, IM—mitral insufficiency, IT—tricuspid insufficiency, IVS—intraventricular septum, Hb—hemoglobin, Hct—hematocrit, HDL—high-density lipoprotein, IU—units, L—liter, LAD—left-atrial diameter, LDL—low-density lipoprotein, LMCA—left main coronary artery, LV—left ventricular, LVEDD—left-ventricular end-diastolic diameter, LVEF—left-ventricular ejection fraction, mIU—microunits, mg—milligram, mmol—millimole, mm—millimeter, MLR—monocyte-to-lymphocyte ratio, NLR—neutrophil-to-lymphocyte ratio, *n*—number, Plt—platelet count, RVEDD—right-ventricular end-diastolic diameter, TSH—thyroid-stimulating hormone, and WBC—white blood cell count.

**Table 3:** Metabolomic results regarding acetyl-carnitine plasma concentrations.

Parameters Carnitine-Type Serum Concentrations [µM]	Whole Group *n =* 41	Group 1 CAD Group *n =* 25	Group 2 Non-CAD Group *n* = 16	*p* Group 1 vs. 2
CO-L-Carnitine	43.60 (38.80–53.90)	43.40 (36.20–57.50)	44.65 (39.48–48.90)	0.873
C2 L-Acetyl-carnitine	8.56 (6.49–10.10)	7.49 (4.79–9.23)	9.36 (8.57–10.23)	0.009 *
C3 ** Propionyl-carnitine	0.40 (0.31–0.50)	0.40 (0.00–0.51)	0.00 (0.00–0.49)	0.807
C3-DC(C4-OH) Malonyl-carnitine **	0.07 (0.00–0.09)	0.07 (0.00–0.09)	0.07 (0.00–0.09)	0.579
C4 Isobutyryl-L-carnitine/Butyryl-carnitine	0.23 (0.23–0.33)	0.23 (0.18–0.33)	0.23 (0.21–0.32)	0.936
C4:1 Butenyl-carnitine	0.04 (0.04–0.05)	0.04 (0.04–0.05)	0.04 (0.03–0.05)	0.640
C5 2-Methylbutyroyl-carnitine/Isovaleryl-carnitine/Valeryl-carnitine/Pivaloyl-carnitine	0.15 (0.13–0.19)	0.15 (0.12–0.20)	0.15 (0.13–0.20)	0.936
C5-DC (C6-OH) Glutaryl-carnitine	0.04 (0.03–0.05)	0.04 (0.03–0.04)	0.05 (0.04–0.06)	0.164
C5-OH (C3-DC-M) Hydroxyvaleryl-carnitine/Methylmalonyl-carnitine	0.08 (0.07–0.08)	0.08 (0.07–0.08)	0.07 (0.07–0.08)	0.267
C8 Octanoyl-carnitine **	0.00 (0.00–0.25)	0.00 (0.00–0.21)	0.22 (0.00–0.27)	0.086
C10 Decanoyl-carnitine **	0.31 (0.00–0.40)	0.00 (0.00–0.37)	0.36 (0.19–0.44)	0.040 *
C10:2 2-trans,4-cis-Decadienoyl-carnitine	0.05 (0.04–0.05)	0.04 (0.04–0.05)	0.05 (0.04–0.05)	0.067
C12:1 trans-2-Dodecenoyl-carnitine **	0.19 (0.15–0.22)	0.17 (0.13–0.20)	0.22 (0.18–0.24)	0.008 *
C14:1 cis-5-Tetradecenoyl-carnitine	0.10 (0.08–0.14)	0.10 (0.07–0.13)	0.13 (0.10–0.15)	0.002 *
C14:2 3,5-Tetradecadien-carnitine **	0.03 (0.03–0.05)	0.03 (0.02–0.04)	0.04 (0.03–0.05)	0.043
C18:1 Oleoyl-carnitine/Vaccenyl-carnitine/Elaidic-carnitine	0.17 (0.15–0.19)	0.16 (0.14–0.18)	0.18 (0.17–0.27)	0.007 *
C18:2 Linoelaidyl-carnitine/Linoleyl-carnitine	0.05 (0.04–0.06)	0.05 (0.04–0.06)	0.05 (0.05–0.07)	0.698

* statistically significant. ** the values that did not reach the LOD were corrected to zero. Abbreviations: CO—L-Carnitine; C2—L-Acetyl-carnitine; C3—Propionyl-carnitine; C3-DC(C4-OH)—Malonyl-carnitine; C4—Isobutyryl-L-carnitine/Butyryl-carnitine; C4:1—Butenyl-carnitine; C5 2—Methylbutyroyl-carnitine/Isovaleryl-carnitine/Valeryl-carnitine /Pivaloyl-carnitine; C5-DC (C6-OH)—Glutaryl-carnitine; C5-OH (C3-DC-M)—Hydroxyvaleryl-carnitine/Methylmalonyl-carnitine; C8—Octanoyl-carnitine; C10—Decanoyl-carnitine; C10:2—2-trans,4-cis-Decadienoyl-carnitine; C12:1—trans-2-Dodecenoyl-carnitine; C14:1—cis-5-Tetradecenoyl-carnitine; C14:2—3,5-Tetradecadien-carnitine; C18:1—Oleoyl-carnitine/Vaccenyl-carnitine/Elaidic-carnitine; C18:2—Linoelaidyl-carnitine/Linoleyl-carnitine; *n*—number; µM—micrometer.

**Table 4:** The correlation between acetyl-carnitine and the male CAD group.

Acetylcarnitines	Male CAD Group Rank-Biserial Correlation	95% CI	*p*
C2	0.385	0.047–0.644	0.036 *
C10	0.524	0.219–0.735	0.003 *
C12.1	0.474	0.155–0.703	0.010 *
C14.1	0.467	0.146–0.698	0.011 *
C14.2	0.285	(−) 0.066–0.573	0.123
C18.1	0.388	0.050–0.646	0.35 *

* statistically significant. Abbreviations: C2—L-Acetyl-carnitine; C10—Decanoyl-carnitine; C12:1—trans-2-Dodecenoyl-carnitine; C14:1—cis-5-Tetradecenoyl-carnitine; C14:2—3, 5-Tetradecadien-carnitine; C18:1—Oleoyl-carnitine/Vaccenyl-carnitine/Elaidic-carnitine.

## Data Availability

The data supporting this study’s results will be shared for three years after publication by the corresponding author via e-mail upon making a reasonable request.
